# Instance of a Heteroplasmic Mitogenome in Alvinocaridid Shrimp *Mirocaris fortunata* (Martin & Christiansen 1995) Found at the Moytirra Deep‐Sea High‐Temperature Hydrothermal Vent Field

**DOI:** 10.1002/ece3.73956

**Published:** 2026-07-01

**Authors:** Paola E. Campos, Patrick C. Collins, Aoife Ruane, Jeanette E. Carlsson, Jens Carlsson

**Affiliations:** ^1^ Area 52 Research Group, School of Biology & Environment Science and Earth Institute University College Dublin Dublin Ireland; ^2^ Queen's University Belfast Marine Laboratory (QML) Portaferry Northern Ireland UK

**Keywords:** alvinocarididae shrimp, heteroplasmy, life in extreme environment, mid‐Atlantic ridge fauna, *Mirocaris fortunata*, mitochondrial genome

## Abstract

In this study, we report the complete mitochondrial genome of the deep‐sea hydrothermal vent shrimp *Mirocaris fortunata* (Alvinocarididae) from shotgun sequencing data on an individual tail tissue. The 15,923‐bp‐long sequence displays 98.72% pairwise identity with its closest relative, *Mirocaris indica*. A significant proportion of the mitochondrial genome (0.63%) corresponds to heteroplasmic sites that were found on 14 of the 37 genes, including *cox1*, though all such sites induce synonymous mutations. This level of heteroplasmy may serve as the first step for recombination of the mitogenome by paternal leakage and/or a less effective purifying selection in somatic tissues. We also take advantage of the shotgun deep sequencing strategy to assess the metagenomic composition of the sample and are able to detect other deep‐sea hydrothermal vent species present at the vent system.

## Graphical abstract

First complete mitochondrial genome of the deep‐sea hydrothermal vent shrimp *Mirocaris fortunata* (Alvinocarididae) from shotgun sequencing data on an individual tail tissue. The 15,923‐bp‐long sequence displays 98.72% pairwise identity with its closest relative, *Mirocaris indica*. A significant proportion of the mitochondrial genome (0.63%) corresponds to heteroplasmic sites that were found on 14 of the 37 genes, including *cox1*, though all such sites induce synonymous mutations.

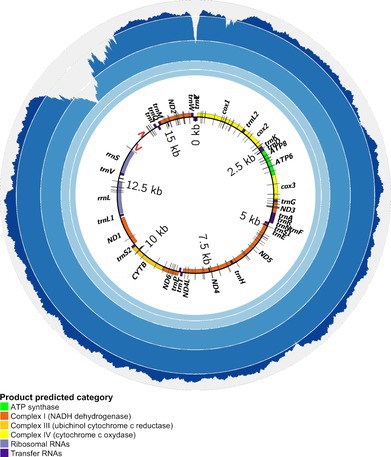

## 1 Introduction

*Mirocaris* Vereshchaka, 1997 is a genus of deep‐sea hydrothermal vents‐endemic shrimp of the Alvinocarididae family. It is morphologically distinguishable from the other genera of this family (Komai and Segonzac 2003). The genus contains two species: the species type *Mirocaris fortunata* (Martin and Christiansen 1995), first described at the Lucky Strike point in the Mid‐Atlantic Ridge (MAR) under the now revised name *Chorocaris fortunata* (Martin and Christiansen 1995), and *Mirocaris indica* (Komai et al. 2006) on the Central Indian Ridge near the Rodriguez Triple Junction (Komai et al. 2006). Both species are morphologically distinguished by the setation of the chela of the first pereopod, suggesting a different diet, with the presence of the trait in *M. fortunata* allowing it to efficiently pick and feed on particles directly from the substrate (Gebruk et al. 2000; Komai et al. 2006).

*M. fortunata* is found on the actively venting sulphide structures of hydrothermal vent fields along the MAR between 12° N and 45° N (Bonnivard et al. 2009; Fabri et al. 2011), with the Moytirra site, discovered in 2011 (Wheeler et al. 2013), as its most northern presence recorded (Collins et al. 2020). *M. fortunata* habitat is spanning high pressure (from 8 MPa at the Menez Gwen site to 42 MPa at Ashadze) and temperature gradients (4°C–25°C) (Shillito et al. 2006; Bonnivard et al. 2009) even though *M. fortunata* is usually found in microhabitats at 10°C in communities as dense as 80–90 individuals per m^2^ (Martin and Christiansen 1995; Gebruk et al. 2000). Living in a chemosynthetic environment, *M. fortunata* has been observed feeding on free‐living bacteria and sulphur‐oxidising bacterial mats on the vent surfaces and from tissues of dead invertebrates and mussel beds (Gebruk et al. 2000; Komai et al. 2006). This diet may be the reason for the genus endemism to deep‐sea hydrothermal vent as the bacteria they feed on are specialised to highly reduced hydrothermal vent fluids (Orcutt et al. 2011).

Deep‐sea hydrothermal vent chimneys are unstable, hypervariable environments with high variability in temperature, pressure, heavy metals and sulphide over very short spatial and temporal scales (Sarradin et al. 1999), making for an extreme, chemosynthetic environment. Moytirra displays an uncommon setting and isolated location compared to the other active high‐temperature hydrothermal vent sites in the North Atlantic (Wheeler et al. 2013). Indeed, at 45°28.4′N, 27°50.7′W and at a depth of 2845–3060 m rising from an axial volcanic ridge, the Moytirra site is tectonically controlled, heated by an off‐axis magma body, and purely basalt‐hosted but uniquely situated midway up the median valley wall (for full site description, see Wheeler et al. (2013)).

In this work, we report the full mitochondrial genome (mitogenome) sequence of *M. fortunata*, displaying heteroplasmic sites on 0.63% of its mitogenome, from next generation sequencing data on tail tissue of a Moytirra sample. This may be used for future population genetic and phylogeographic analyses of the Alvinocarididae with more informative and determining molecular markers. We propose that the heteroplasmic mitogenome may act as a reservoir of mutations available to better adapt to the high‐stress, hypervariable environment *M. fortunata* evolves in. We also study the metagenomic composition of the sample and show that this, together with the mitogenome sequence, may have application for identifying environmental DNA biomarkers of active hydrothermal vents in the Mid‐Atlantic Ridge.

## 2 Material and Methods

### 2.1 Sampling

Sampling was undertaken between 11 July and 14 August 2011 at 3000 m water depth at the Moytirra hydrothermal vent field (45°28.4′N, 27°50.7′W) in the Mid Atlantic Ridge using the suction device of the remotely operated vehicle Holland I launched from the research ship RV Celtic Explorer during the VENTuRE survey (see Wheeler et al. (2013) for a detailed site description). The *Mirocaris* were found in aggregations around actively venting sulphide structures. Individuals were morphologically identified on site and preserved in molecular grade ethanol at −20°C until DNA extraction.

### 2.2 DNA Extraction, Quality Control and PCR

DNA was extracted from specimen ‘S3’ using Chelex and Proteinase K (Sepp et al. 1994) from ~1.5 mg of tail tissue following cleaning of the tissue with deionised water. Quality assessment of the extract was performed for purity and concentration with BioDrop μLite+ (Harvard Bioscience) microvolume spectrophotometer.

### 2.3 Library Preparation and Sequencing

Library preparation and shotgun sequencing were outsourced to Novogene (https://www.novogene.com/eu‐en/). Briefly, genomic DNA was randomly sheared into shorter fragments, end‐repaired, A‐tailed and converted into a double‐stranded library using custom TruSeq protocol and thus sequenced on Illumina 25‐B platforms in a paired‐end 2 × 150 cycles configuration on a single lane of the NovaSeq X+ flow cell.

### 2.4 Sequencing Data Initial Processing

Artefactual homopolymer sequences with an entropy below 0.6 were removed using BBDuk from BBMap 38.34 (Joint Genome Institute 1997). Illumina adaptors were trimmed out using the Illuminaclip option in Trimmomatic 0.39 (Bolger et al. 2014). Additional quality‐trimming was performed with Trimmomatic 0.39 based on base‐quality (LEADING:15; TRAILING:15; SLIDINGWINDOW:5:15) and read length (MINLEN:30). Paired reads were then merged using AdapterRemoval 2.3.4 (Schubert et al. 2016) and either used as they were for mitogenome reconstruction or metagenomic analysis.

### 2.5 Mitogenome Reconstruction and Characterisation

*Mirocaris fortunata* mitochondrial DNA (mtDNA) sequence was reconstructed by mapping quality‐trimmed reads to *M. indica* mitogenome reference sequence (NC_054368.1) using Bowtie 2 (options ‐‐non‐deterministic ‐‐very‐sensitive) (Langmead and Salzberg 2012). PCR duplicates were removed using picard‐tools 3.2.0 MarkDuplicates (Broad Institute 2018). Subsequent sequencing depths (number of mapped reads at each base of the sequence) were computed using BEDTools genomecov 2.31.1 (Quinlan and Hall 2010) and coverage (proportion of the sequence covered at 1*X* depth) was calculated with R. The presence of a coding DNA sequence (CDS) was assumed when its sequence coverage was found above a 75% threshold. Single nucleotide polymorphisms (SNPs) were called with GATK 4.2.0.0 VariantFiltration (DePristo et al. 2011). They were considered dubious and filtered out if they met either of the following conditions: ‘depth < 30*X*’, ‘mapping quality < 30’; and were flagged as heteroplastic if they displayed a ‘minor allelic frequency ≥ 0.1’. Consensus mitogenome sequence was then constructed by introducing the remaining passing, high‐quality SNPs in *M. indica* mitogenome sequence and replacing filtered‐out variants and uncovered positions by an ‘n’; heteroplastic sites were coded as the symbol for the two, three (or four in a single case) bases if they were found with a frequency above 0.1 at the position. All steps were then reiterated on the consensus sequence and linkage between heteroplastic sites was checked within a 150 bp sliding window. *De novo* annotation of the sequence was performed using MITOS (Bernt, Donath, et al. 2013) and the structure of the gene products were predicted with AlphaFold3 (Abramson et al. 2024) online server. Annotations were manually curated so positions fitted those of *M. indica* mitogenome sequence (NC_054368.1) when pTM (predicted template modelling) ratios of homologue genes were inferior to 0.95 and found within 5 bp of each other. Secondary structure of transfer RNA was predicted with RNAstructure (Reuter and Mathews 2010) online server with default parameters. Depths, gene annotations and heteroplastic sites were graphically represented with CIRCOS 0.69.9 (Krzywinski et al. 2009).

### 2.6 Metagenomics

The metagenomic composition of the sample was performed by comparing reads against the NCBI nucleotide database (January 2025) with the blastn command line of NCBI BLAST 2.12.0+ (Altschul et al. 1990). Only top hits of length above 75 bp with an e‐value below 0.001 were saved. Hits to *Alvinocaridinides* spp., *Alvinocaris* spp., *Chorocaris* spp., *Mirocaris* spp. or *Rimicaris* spp. were grouped into a single category as unaccepted (*Alvinocaridinides*) or junior synonyms of *Mirocaris* or *Rimicaris* (*Chorocaris*) or mislabelled (*Rimicaris fortunata* instead of *M. fortunata*) sequences, or misassigned (*Alvinocaris*, outside of geography range—GBIF https://www.gbif.org/species/4473). Only *Mirocaris* or *Rimicaris* spp. shrimps within the Alvinocarididae family were reported at the Moytirra vent field (Wheeler et al. 2013).

## 3 Results

### 3.1 DNA and High‐Throughput Sequencing Data Quality

Sample yielded 46.9 ng DNA per microliter from ~1.5 mg of tail tissue. The sequencing generated 7.2 Gb of sequences, corresponding to 48.7 M paired‐end reads with a mean base call accuracy of 99.98%.

### 3.2 Mitogenome Reconstruction and Characterisation

**FIGURE 1:**
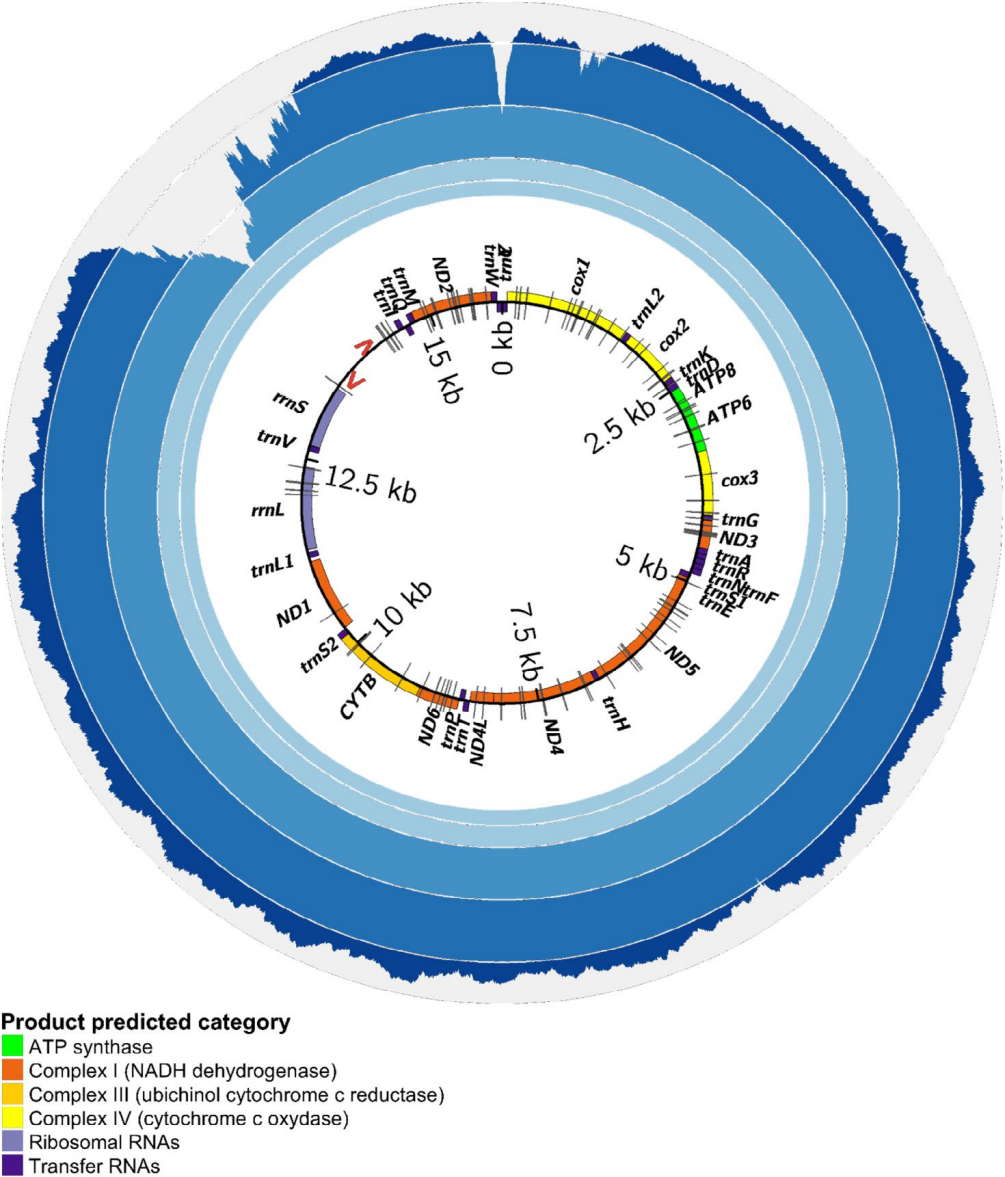
*Coverage plot of reconstructed Mirocaris fortunata annotated mitogenome sequence*. From inside to outside, a light to dark blue scale (delimited by a white line) represents 1, 1–5, 5–30, 30–50, 50–115‐fold depth. Heteroplastic sites are indicated (grey lines) on the annotated sequence displaying genes (coloured by product category) encoded on the light (inner position) or heavy strand (outer position) whose origin of replication is indicated on the respective strand (red arrows).

*M. fortunata* complete mtDNA sequence was reconstructed and submitted to GenBank (accession number PX614637). The total length of the circularised sequence was 15,923 bp as no gap nor uncovered position was observed with coverage (proportion of sequence covered) at 30*X* of 98.0% and a mean depth of 88.5*X* (Figure 1). *M. fortunata* mitogenome displayed 98.72% pairwise identity with *M. indica* mitogenome (204 SNPs for a 15,924 nt alignment, including an insertion of 2 bp and a deletion of 1 bp) and shared all 36 identified genes in a similar sequence arrangement, with the addition of one transfer RNA (*trnD*‐gtc) not reported on *M. indica* sequence (Table 1), with 23 of these 37 genes on the heavy strand. Nine of the 13 coding sequence (CDS) genes were found on the heavy strand (all but *ND1*, *ND4*, *ND4L* and *ND5*). All but one CDS gene started with typical ATN start codon, with *ND5* as the exception. Transfer RNA genes sequence length varied from 63 bp (*trnR*‐cga) to 73 bp (*trnS2*‐tga). All 22 tRNAs (one for each amino acid, two for Leucine and two for Serine) but *trnS1*‐tct, *trnH*‐cac, *trnQ*‐ttg, *trnW*‐tca and *trnC*‐gca displayed typical cloverleaf‐shaped secondary structure and L‐shaped tertiary structure. Intergenic regions were 1 bp to 215 bp long with the origin of replication within a 1078‐bp‐long region. The overall base composition of *M. fortunata* mitogenome was 34.5% A, 21.5% C, 11.2% G and 31.9% T, with 0.2% R, 0.4% Y, 0.3% N and under 0.1% of H, K and W, accounting for a GC content of 33.0%. There were 101 sites displaying multiple alleles (0.63% of the mitogenome, Table S1), 86% of them displayed a minor allelic frequency above 0.25 (all but 14/101, of which two sites had either 3 or 4 alleles at frequency above or equal to 0.10) and 45 with a minor allelic frequency between 0.45–0.50. There were 84 of these 101 multiallelic sites on genes *atp6*, *cox1*, *cox2*, *cox3*, *cytb*, *ND1*, *ND2*, *ND3*, *ND4*, *ND4L*, *ND5*, *ND6*, six in RNA genes *rrnL* and *trnG*‐gga, and the remaining 11 in two intergenic regions. They are spaced from each other by 2 to 1572 bp (with a mean of 158 ± 226 bp), with an apparent higher frequency between the 14,107 bp (origin of replication of the heavy strand) and 9024 bp clockwise (end of *ND6*) than between 9024 bp and 13,715 bp (origin of replication of the light strand) with a mean space of 120 ± 124 bp and 412 ± 489 bp, respectively. Low depth (< 30*X*) between 13,584 bp and 14,114 bp does not allow for the identification of high confidence SNPs or heteroplastic sites. Most heteroplasmic sites displayed transversions (89.11%) with C‐T variations as the most common (68.89% of transversions). All heteroplasmic sites impacted either 3rd (72/101) or 1st (12/101) position in codon and displayed synonymous translations (dN/dS = 0).

**TABLE 1:** *Mitogenome organisation of Mirocaris fortunata*.

Gene	Start position	Stop position	Length	Intergenic region length	Strand	*N* heteroplasmic sites in locus	Starting codon
trnY‐gta	1	65	65	0	−	0	
cox1	63	1599	1537	−3	+	13	ATC
trnL2‐taa	1599	1663	65	−1	+	0	
cox2	1668	2378	711	4	+	5	ATG
trnK‐ttt	2358	2425	68	−21	+	0	
trnD‐gtc	2444	2511	68	18	+	0	
ATP8	2512	2670	159	0	+	0	ATT
ATP6	2664	3338	675	−7	+	7	ATG
cox3	3338	4126	789	−1	+	3	ATG
trnG‐tcc	4129	4193	65	2	+	1	
ND3	4200	4547	348	6	+	6	ATA
trnA‐tgc	4548	4611	64	0	+	0	
trnR‐tcg	4612	4674	63	0	+	0	
trnN‐gtt	4677	4741	65	2	+	0	
trnS1‐tct^a^	4742	4809	68	0	+	0	
trnE‐gta	4810	4878	69	0	+	0	
trnF‐gaa	4879	4945	67	0	−	0	
ND5	4947	6674	1728	1	−	15	GTG
trnH‐cac^a^	6675	6738	64	0	−	0	
ND4	6740	8077	1338	1	−	8	ATG
ND4L	8070	8370	301	−8	−	2	ATG
trnT‐tgt	8375	8441	67	4	+	0	
trnP‐tgg	8442	8506	65	0	−	0	
ND6	8509	9024	516	2	+	6	ATT
CYTB	9024	10,160	1137	−1	+	4	ATG
trnS2‐tga	10,158	10,230	73	−3	+	0	
ND1	10,247	11,186	940	16	−	1	ATA
trnL1‐tag	11,239	11,305	67	52	−	0	
rrnL	11,342	12,398	1057	36	−	5	
trnV‐tac	12,612	12,677	66	213	−	0	
rrnS	12,682	13,477	796	4	−	0	
trnI‐gat	14,556	14,623	68	1078	+	0	
trnQ‐ttg^a^	14,641	14,709	69	17	−	0	
trnM‐cat	14,722	14,788	67	12	+	0	
ND2	14,795	15,790	996	6	+	14	ATA
trnW‐tca^a^	15,788	15,858	71	−1	+	0	
trnC‐gca ^a^	15,857	15,923	67	−2	−	0	

*Note:* Genes label, gene start and end positions, length of gene (bp), length of intergenic regions (bp), strand encoding gene and starting codon sequence are indicated for the 37 genes of the mitochondrial genome of the *M. fortunata* sample.

^a^ tRNAs without typical cloverleaf‐shaped secondary structure nor L‐shaped tertiary structure.

### 3.3 Metagenomics

**FIGURE 2:**
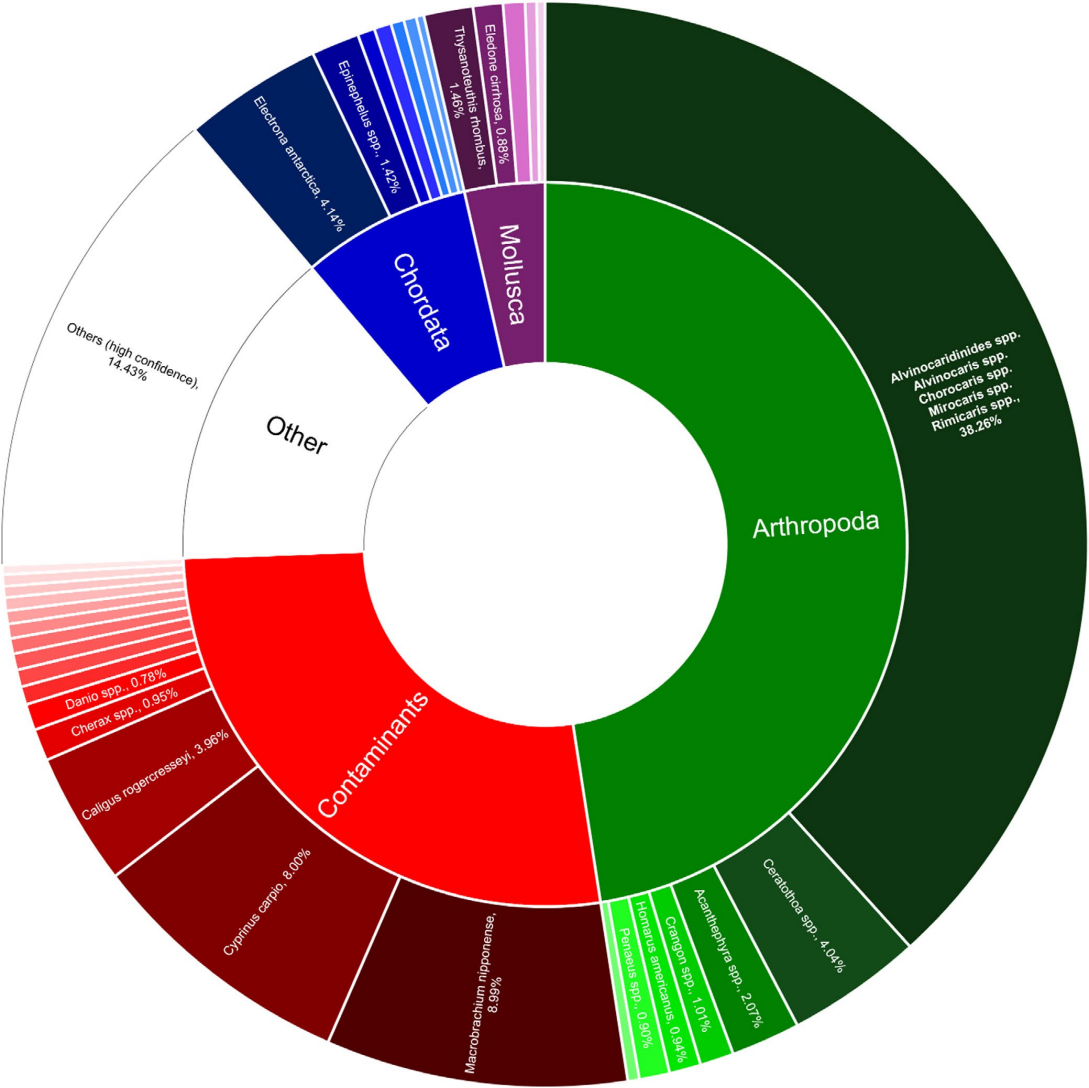
*Metagenomic composition of* Mirocaris fortunata *tail tissue sample*. Proportions and numbers of total reads classified as unassigned reads (‘dark matter’, not shown on graphic): 97.92% (47,685,985 reads), low‐confidence taxonomically identified reads (not shown on graphic): 1.18% (572,667 reads), high‐confidence taxonomically identified reads: 0.91% (440,952 reads). Proportions of high‐confidence taxonomically identified reads assigned to Arthropoda (green): 47.58%, Chordata (blue): 7.58%, Mollusca (purple): 3.58%, Other phyla (white, genera represented by less than 1000 reads): 14.43%, Contaminants (red): 26.84%. Within these groups, taxa are identified to the species level and aggregated by genus when more than one species per genus is found.

Sequences confidently assigned taxonomically mostly belonged to phylum Arthropoda (47.58%) with reads mapping to *Alvinocaridinides* spp., *Alvinocaris* spp., *Chorocaris* spp., *Mirocaris* spp. and *Rimicaris* spp. sequences accounting for most of it (38.26% of confidently assigned reads). Other phyla were also identified in the sample, such as Chordata (7.58% with *Electrona antarctica* 4.14%, *Epinephelus* spp. 1.42%…), Mollusca (3.58% with *Thysanoteuthis rhombus* 1.46%, *Eledone cirrhosa* 0.88%, *Bathymodiolus* spp. 0.35%…) as well as other species of Arthropoda (*Ceratothoa* spp. 4.04%, *Crangon* spp. 1.01%…). Allochthonous, non‐marine species were also identified, accounting for 26.84% of all confidently assigned reads (Figure 2; Table S2). Genera supported by less than 1000 reads each represented 14.43% of these reads (2199 genera found). However, even though DNA of the specimen was extracted from tail tissue and thus expected to be mostly belonging to *M. fortunata*, all confidently assigned reads represented only 0.91% of all reads produced, with another 1.18% of the reads assigned with low confidence and 97.92% of the reads not assigned to any known taxa.

## 4 Discussion

We successfully reconstructed the first complete mitogenome sequence for *M. fortunata* from the MAR Moytirra site. This novel result may be used to develop specific primers targeting informative markers, enable comparative and evolutionary genomics, assess the population genetic structure and perform phylogenetic and phylogeographic analyses on whole mitogenome data to get new insights into the speciation and deep‐sea thermal vents systems colonisation by *Mirocaris*.

Indeed, the characteristics of the mitogenome (i.e., predominantly uniparentally transmitted, short circular, non‐recombining sequence with a small conserved gene repertoire displaying variable regions (e.g., displacement loop of the control region) flanked by highly conserved regions resulting in a relatively high evolution rate) (Bernt, Braband, et al. 2013) make it a commonly used and suitable marker for DNA barcoding and population genetic studies based on molecular diversity (Galtier et al. 2009). However, many exceptions to these assumptions have been recorded in plants (Kmiec et al. 2006) as well as in animals (Piganeau et al. 2004) such as squamates (Ujvari et al. 2007), birds (Gandolfi et al. 2017), fish (Guo et al. 2006; Sun et al. 2021), mammals (Vollmer et al. 2011; Payne et al. 2013), bivalves (Boyle and Etter 2013; Ghiselli et al. 2019; Martínez et al. 2023), insects (Magnacca and Brown 2010; Kaczmarczyk‐Ziemba et al. 2024), crustaceans (Williams et al. 2017; Rodríguez‐Pena et al. 2020; Chow et al. 2021; Iketani et al. 2021; Ho and Mohd Hanafiah 2024), among which a transpecific, stable, inherited heteroplasmy dating back 30 million years at least was identified (Doublet et al. 2008). These recent works demonstrated how heteroplasmy (mitogenome state where multiple haplotypes coexist instead of a singular one, caused by somatic mutagenesis during an individual's lifetime or by paternal leakage during fecundation) could be extensively studied thanks to next generation sequencing technologies with a new robustness in its detection using deep sequencing, a property lacking in first generation sequencing like Sanger. Indeed, though it displays the lowest base calling error of all sequencing technologies to date, it also relies on human curation for biallelic bases such as seen in heteroplasmy that would most of the time be labelled sequencing error. Such behaviour perpetrated by geneticists for decades may have postponed the identification of heteroplasmy in many species across the tree of life that is now slowly being reevaluated. Our results show that, just like some other crustaceans, *M. fortunata* displays heteroplasmy. Heteroplasmy may obscure the expected genetic signal needed for molecular identification or population genetics and result in incorrect taxonomic conclusions by affecting accurate species delimitation, overestimating species richness and underestimating effective population size based on haplotype diversity (as, in the presence of heteroplasmy, there are more haplotypes than individuals).

While S3 *M. fortunata* mitogenome is heteroplasmic, it presents the usual mitochondrial gene repertoire with the sequence itself 98.72% similar to *M. indica* 's and all mutations of heteroplastic sites are synonymous. The heteroplasmy should therefore have no effect on the respiratory chain metabolism and survival of the individual and may theoretically be transmitted to offspring. Although the stability of heteroplasmy in *M. fortunata* at the population or species level and across generations was obviously not tested given the study sampling size of one specimen, synonymous mutations are not expected to disappear. Indeed, the frequency of non‐synonymous mutations, compared to synonymous ones, has been shown to decrease over a few generations, therefore preventing deleterious mutations from passing from one generation to the other (Stewart et al. 2008), whereas too harmful mutations result in unviable organisms and may not be observed in the population at all if lethal. While this may indicate that purifying selection is not counter‐selecting mitogenomes displaying these synonymous mutations, heteroplasmy can be detrimental by itself as seen in mice, impacting their respiratory exchange ratio, behaviour and cognitive abilities (Sharpley et al. 2012).

However, we argue that this is not the case in S3 and that the maintenance of heteroplasmy at low level may serve as a reservoir of multiple haplotypes as the first step for recombination to overcome Muller's ratchet (Muller 1964) for *M. fortunata*. Indeed, under Muller's ratchet, non‐recombining genomes would indefinitely accumulate deleterious mutations, resulting in a too high genetic load and the extinction of the population. Overcoming Muller's ratchet effect may be achieved through a low rate of paternal leakage during fecundation of the oocyte by the sperm (Greiner et al. 2015), but may also be the result of a less effective purifying selection in somatic tissues (Wilton et al. 2018). While this latter process may affect all types of genomes within a cell, it may lead, in the case of the mitogenome, to a higher accumulation of spontaneous mutations given its proximity to intracellular oxidative free radicals and its relatively limited protection and repair mechanisms (Wallace 2007). This may further be exacerbated by DNA damaging factors, such as found in the highly ecotoxic and slightly radioactive environment in which *M. fortunata* lives. Therefore, the multiple haplotypes co‐existing within an individual would form a capital of sequences on which the apparition of deleterious mutations would not immediately result in disease by compensating the phenotype through alternative products. This sequence polymorphism is not the only one reported in *M. fortunata*: its nuclear genome size is variable (more than 1.5‐fold, with two distinct classes of lengths) within and between MAR populations and such variation could not be attributed to ploidy changes (Bonnivard et al. 2009). Genetic drift in the *M. fortunata* mitogenome might be somewhat compensated, due to sequence polymorphisms, which may be important adaptive traits given its endemism to the localised and short‐lived hydrothermal vent system and hazardous dispersal and colonisation capability (Mullineaux 2014), especially at the uniquely situated and isolated location of Moytirra (Wheeler et al. 2013). These interpretations remain entirely speculative as they are extrapolated from the observation of a single specimen and will need to be tested in future genetic studies at population (across MAR sites) and species level to bring any conclusion on the adaptive power of *M. fortunata*. Yet, its close relative, *Rimicaris exoculata*, has been shown to present high genetic variability and heteroplasmy at the populations scale across five localities of the MAR system, with an increase of haplotype diversity over time and generations in some of them (Teixeira et al. 2011), but very low genetic variability from alloenzyme data compared to hydrothermal vent species (Creasey et al. 1996). In these same localities spanning 7100 km, *Rimicaris exoculata* has also been found to lack spatial genetic structure (Teixeira et al. 2011, 2012) and no genetic differentiation of populations, revealing large‐scale effective dispersal across the highly‐fragmented hydrothermal vent sites (Teixeira et al. 2012). *M. fortunata* may display similar trends that remain to be tested.

While these sequence polymorphisms (mtDNA heteroplasmy and variable nuclear genome size) may be specific to *M. fortunata*, and the result of adaptation to Moytirra, some more global mechanisms may be involved to explain its adaptability to such an environment, that are shared across taxa endemic to deep‐sea hydrothermal vent systems. Indeed, even though systematic exploration of hydrothermal vent systems across the oceans is not yet realised, it appears that their endemic metazoan taxa are crustaceans, molluscs and annelids (making up ~90% of the total vent fauna (Tunnicliffe et al. 1998; Wolff 2005; Bachraty et al. 2009))– with no vertebrates identified as strictly endemic instead of vagrant visitors but for the deep‐sea fish *Pachycara thermophilum* (Geistdoerfer 1994), found in the Atlantic Ocean and at MAR, and *Symphurus thermophilus* (Munroe and Hashimoto 2008) found punctually on western Pacific ocean seamounts. Although eukaryotes share highly conserved DNA repair mechanisms (Painter 1974; Wood 1996; Kunkel and Erie 2005; Huertas 2010), their efficiency differs, with vertebrates having more rigid a system and strand discrimination by DNA methylation during replication to recognise the parental strand in addition to the core Mismatch‐Mediated Repair proteins. This apparent lesser efficiency in crustaceans, molluscs and annelids compared to vertebrates results in a higher mutation rate that may be beneficial and necessary to evolve, adapt and colonise deep‐sea hydrothermal vents by countless ‘trials and errors’ over time and could explain their high endemicity compared to the anecdotical one of vertebrates. We cannot conclusively state at what level the mtDNA heteroplasmy in *M. fortunata* is occurring (i.e., organelle (mitochondria with multiple alternative mtDNA haplotypes), cell (homoplasmic mitochondria with multiple alternative mtDNA haplotypes) or tissue (mosaic of homoplasmic cells of alternative mtDNA haplotypes)), as this study analysed a single individual and its DNA was extracted from whole tail tissue. Single‐cell, multi‐cell, multi‐tissue and multi‐individual genetic studies would be needed to resolve this uncertainty. We can nonetheless exclude a false heteroplasmy caused by nuclear‐encoded mitochondrial DNA (NUMT), non‐functional copies of mitochondrial sequences dynamically incorporated into the nuclear genome that may retain close similarity of sequence with the original mitochondrial sequences (Bensasson 2001). Indeed, while NUMT fragments are indisputably present in our sequencing data, they can be bioinformatically filtered out by adopting strict mapping parameters, preferably on PCR‐ or capture‐free DNA (Marshall and Parson 2021). Thus, the stringency of the mapping strategy from deep shotgun sequencing data we adopted only allowed for the least divergent NUMT fragments to also map to the mtDNA sequence used during the reconstruction of *M. fortunata* mitogenome. More divergent NUMT fragments, by their older incorporation in the nuclear genome and lack of selection on their products, may be revealed on CDS gene sequences by the presence of non‐synonymous mutations leading to variantly folded or nonfunctional proteins and/or early apparition of stop codons (Song et al. 2008); patterns that are absent in *M. fortunata* reconstructed mitogenome gene content. The NUMT fragments not filtered out during SNP calling, by their little divergence to the mtDNA sequence and lesser frequency compared to the thousand of mitochondrial DNA copies per animal cell (Scheffler 2001), would not affect the base calling of the minor allele in heteroplastic sites as minor allelic frequency needs to be over 0.1 (10% of all reads at the variable position) and depth at the site must be above 30*X*. Furthermore, read depth supporting the reconstructed mtDNA sequence, without large punctual positive deviations from the mean depth, does not favour the hypothesis of multiallelic sites being caused by NUMT fragments. That is, unless almost the whole mtDNA sequence (from 14,114 to 13,584 bp clockwise) has been fully incorporated as a single NUMT segment into the nuclear genome, such as reported in some mammals (Shi et al. 2017; Balciuniene and Balciunas 2019; Lutz‐Bonengel et al. 2021). Read depth falling from 13,584 to 14,114 bp may be the result of the stringent mapping parameters not allowing reads to map if too divergent from the expected sequence, as this region corresponds to the control region with its hypervariable D‐loop, as has been observed on the shrimp *Penaeus* (*Litopenaeus*) *vannamei* heteroplasmic mitogenome (Soares et al. 2021). Strict isolation of mitochondria from the nucleus during DNA extraction using Differential Centrifugation protocol (Boyle and Etter 2013) or a targeted mitogenome rolling circle amplification such as MitoRS (Marquis et al. 2017), for example, would be needed to test empirically the presence of NUMTs in *M. fortunata* in the absence of its nuclear genome sequence or dedicated NUMT database.

These known limitations notwithstanding, the mitogenome is a preferred target for environmental DNA (eDNA) assays, given the high relative abundance in the cell compared to nuclear genome loci and the non‐invasiveness of the sampling method. Designing primers targeting a mitochondrial gene, such as the one coding for the cytochrome oxidase C subunit I (*cox1*), should factor in the heteroplasmic sites and include degenerate sites to account for the variability in the sequence. The control region, another commonly used target region, including crustaceans (Soares et al. 2021), may not be optimal, as discussed above.

In the current study, the adoption of a shotgun deep sequencing strategy allowed not only for the reconstruction of the mitogenome of *M. fortunata* but also for its target‐free metagenomic assessment. It made for the identification of DNA of co‐occurring deep sea species such as hydrothermal vent mussels (*Bathymodiolus* spp.), as well as mesopelagic species such as the diamondback squid ( *Thysanoteuthis rhombus* ) and the curled octopus ( *Eledone cirrhosa* ) or the Antarctic lanternfish ( *Electrona antarctica* ) and large‐eye snaggletooth ( *Borostomias antarcticus* ) whose carcasses *M. fortunata* might move over or opportunistically feed on, as seen in mussels (Gebruk et al. 2000; Fabri et al. 2011). However, no DNA from *Peltospira smaragdina*, a limpet found in abundance at Moytirra (Wheeler et al. 2013), nor the *Sulfurimonas*‐like bacteria constituting the biofilm it grazes on (Collins et al. 2020) was identified. While a metabarcoding approach may be more conventional or robust, the shotgun strategy should not be dismissed as our metagenomic result constitutes a glimpse into the deep‐sea hydrothermal community at Moytirra and expands some species distribution range northward, exhibiting the multifaceted application of molecular studies. Taxonomic identification suffers nonetheless from a disparate and incomplete representation of biodiversity in occurrences, genetic and taxonomic databases such as the Global Biodiversity Information Facility (GBIF) (Yesson et al. 2007) and GenBank/NCBI (Troudet et al. 2017; Keck et al. 2023) it relies on. Indeed, an overwhelming 97.92% of the reads were not assigned to any known taxa during the metagenomic analysis, showing our current lack of knowledge about deep‐sea organisms, including those living in hydrothermal vent environments. Nevertheless, we are confident this ‘dark matter’ may represent a wealth of information for future genetic, medical and biotechnological studies focusing on extremophile organisms and their bioproducts, such as the highly successful and multidisciplinary Virus‐X project (Aevarsson et al. 2021).

## 5 Conclusion

Our study presents the complete mitogenome sequence for *M. fortunata* from the MAR Moytirra site and assesses its general organisation. The existence of heteroplasmic sites with high minor allelic frequency (0.1 to 0.5) along the sequence suggests the presence of mechanisms maintaining multiple mtDNA haplotypes within an individual without notable deleterious effects. While this study is profoundly limited by its sampling size, the peek into *M. fortunata* genetic data hints at evolutionary mechanisms that may be involved in its deep‐sea hydrothermal vent endemism, such as a possibly less effective purifying selection of mutations and paternal leakage. As these suppositions remain hypothetical, broader studies at the population and species levels aiming to reconstruct the propagation, adaptation and evolutionary history of *Mirocaris* spp. along MAR sites would be needed to draw further conclusions.

Deep sea environments and their endemic biodiversity, by their fastidious and expensive sampling, remain understudied. While methodological biases exist, shotgun sequencing strategies may constitute an attempt to bridge biodiversity knowledge gaps.

## Author Contributions

**Paola E. Campos:** conceptualization (equal), formal analysis (lead), investigation (lead), methodology (lead), writing – original draft (lead), writing – review and editing (equal). **Patrick C. Collins:** conceptualization (equal), data curation (equal), resources (equal), writing – review and editing (equal). **Aoife Ruane:** conceptualization (supporting), writing – review and editing (supporting). **Jeanette E. Carlsson:** data curation (lead), resources (supporting), writing – review and editing (equal). **Jens Carlsson:** conceptualization (lead), funding acquisition (lead), investigation (supporting), resources (equal), supervision (lead), validation (lead), writing – review and editing (equal).

## Funding

This work was supported by SFI Research Centre for Energy, Climate and Marine (12/IP/1308), the Marine Institute, the Irish Government, Beaufort Marine Research Award in Fish Population.

## Conflicts of Interest

The authors declare no conflicts of interest.

## Supporting information


**Table S1:** Heteroplasmic site characteristics on *Mirocaris fortunata* mitogenome.

**Table S2:** Metagenomic composition of *Mirocaris fortunata* tail tissue sample.

## Data Availability

The authors made available all data presented in this study. Raw Illumina sequencing data has been deposited to the NCBI Sequence Reads Archive under BioProject accession number PRJNA1365054 while *Mirocaris fortunata* mitogenome sequence is found on NCBI GenBank under BankIt accession number PX614637.
